# N-acetylcysteine for youth cannabis use disorder: randomized controlled trial main findings

**DOI:** 10.1038/s41386-025-02061-y

**Published:** 2025-02-05

**Authors:** Kevin M. Gray, Rachel L. Tomko, Nathaniel L. Baker, Erin A. McClure, Aimee L. McRae-Clark, Lindsay M. Squeglia

**Affiliations:** 1 Department of Psychiatry and Behavioral Sciences, Medical University of South Carolina, Charleston, SC USA; 2 Department of Public Health Sciences, Medical University of South Carolina, Charleston, SC USA; 3 Hollings Cancer Center, College of Medicine, Medical University of South Carolina, Charleston, SC USA

**Keywords:** Outcomes research, Psychiatric disorders

## Abstract

Cannabis use disorder is particularly prevalent and impairing among young people, and evidence-based treatments are limited. Prior trials of N-acetylcysteine, added to contingency management as a platform behavioral intervention, yielded positive findings in youth but not in adults. This trial sought to rigorously evaluate whether N-acetylcysteine is efficacious in youth when not paired with a robust behavioral treatment platform. Treatment-seeking youth with cannabis use disorder (*N* = 192, ages 14–21) were randomized to receive a double-blind 12-week course of oral N-acetylcysteine 1200 mg or placebo twice daily; all received weekly medical management and brief behavioral counseling. The primary efficacy outcome was the proportion of negative urine cannabinoid tests during treatment, compared between groups. An array of self-report and urine testing measures were examined secondarily to assess cannabis use reduction and cessation outcomes. The N-acetylcysteine and placebo groups did not differ in proportion of negative urine cannabinoid tests (RR = 0.93, 95% CI = 0.53, 1.64; *p* = 0.80) or self-reported cannabis abstinence (RR = 1.02, 95% CI = 0.63, 1.65; *p* = 0.93) during treatment. The mean percentage of cannabis use days and grams of cannabis used per using day decreased over time during treatment but did not differ between groups. More N-acetylcysteine than placebo treated participants reported gastrointestinal adverse events (63/98 versus 37/94, χ^2^_1_ = 11.9 *p* < 0.001); adverse events were otherwise similar between groups. Findings indicate that N-acetylcysteine is not efficacious for youth cannabis use disorder when not paired with contingency management, highlighting the potentially crucial role of a robust behavioral treatment platform in facilitating prior positive efficacy findings with N-acetylcysteine.

Trial Registration: Clinicaltrials.gov identifier NCT03055377

## Introduction

While cannabis use is increasingly prevalent across age groups, adolescents and young adults represent an age range of particular concern regarding cannabis-related adverse outcomes [1]. Adolescent-onset cannabis use is more than twice as likely as adult-onset use to progress to an impairing pattern of use defined as cannabis use disorder [2]. Moreover, youth who use cannabis regularly are particularly prone to adverse educational, occupational, and mental health outcomes associated with cannabis use [3, 4].

The current evidence base for addressing cannabis use disorder in youth includes psychosocial, behavioral, and family-based interventions [5, 6]. While this array of interventions may be helpful for many young people presenting with cannabis use disorder, effect sizes are small to modest and long-term outcomes are limited. Efforts are afoot to yield improved outcomes, both via bolstering these interventions and via examination of potential pharmacological approaches to complement them. To date, there are no United States Food and Drug Administration approved medications for cannabis use disorder in adolescents or adults. However, amid increased rates of cannabis use disorder, there has been a focus on developing and testing candidate medications for this condition [7, 8].

Among candidate medications for cannabis use disorder is N-acetylcysteine, a compound that has demonstrated amelioration of substance use-induced dysregulation of the neurotransmitter glutamate in the nucleus accumbens in rodent models, as well as associated reductions in substance self-administration [9]. Given that N-acetylcysteine is readily available as an over-the-counter supplement and has demonstrated tolerability across age groups even at high doses when administered to address acetaminophen toxicity, it has been considered a medication with strong potential for broad dissemination and implementation if preclinical findings translate to human substance use disorders [10].

A prior randomized, placebo-controlled trial in youth ages 14–21 evaluated N-acetylcysteine added to brief weekly counseling and a twice-weekly contingency management intervention, in which visit attendance and negative urine cannabinoid test results were monetarily reinforced [11]. Participants receiving N-acetylcysteine had more than double the odds, compared to placebo participants, of achieving cannabis abstinence reflected in negative urine cannabinoid tests. A subsequent similarly designed trial in adults ages 18–50 yielded null findings, with no difference in cannabis use outcomes between N-acetylcysteine and placebo groups, suggesting that N-acetylcysteine’s effect on cannabis use disorder may be developmentally specific to youth [12]. This assertion was further supported by a post hoc analysis of participants ages 18–21 in the adult trial, indicating an effect size favoring N-acetylcysteine over placebo comparable to that observed in the prior youth-focused trial [12]. These discrepant findings across youth versus adult participants may potentially reflect differential effects of N-acetylcysteine based on developmental stage, or may be owing to developmental differences in the course, context, and phenomenology of cannabis use disorder. Of note, given that these trials included contingency management as a robust platform behavioral intervention, questions remained regarding the context in which N-acetylcysteine might be effectively delivered to youth in clinical practice. Specifically, it was unclear whether N-acetylcysteine would yield a positive effect if not paired with contingency management. This is an important consideration, particularly given prior evidence of synergy between pharmacotherapy and contingency management in interventions for youth substance use disorders [13].

The present trial was conducted to evaluate N-acetylcysteine’s efficacy for youth cannabis use disorder when paired with brief medical clinician-delivered cessation counseling and medical management. Findings were deemed relevant for clinical practice, particularly to distinguish whether contingency management—included in a prior youth trial with positive findings, but not in the present trial—is a necessary platform treatment to facilitate N-acetylcysteine’s efficacy for youth cannabis use disorder.

## Participants and methods

### Study design and participants

This randomized, double-blind, placebo-controlled, parallel-group study included a screening period of up to 4 weeks, a 12-week treatment course, and follow-up through week 26 from randomization, with post-treatment visits at approximately weeks 16 and 26. The study received Medical University of South Carolina Institutional Review Board approval and was conducted in accordance with the Declaration of Helsinki and the International Council for Harmonisation of Technical Requirements for Pharmaceuticals for Human Use and Good Clinical Practice guidelines. Study outcomes were pre-registered on clinicaltrials.gov (NCT 03055377). The 2400 mg/day dosage of oral N-acetylcysteine, administered as 1200 mg twice daily, was selected based on prior positive findings with this dosage combined with contingency management [11]. Participants ages ≥18 provided written informed consent. Written parental consent and participant assent were obtained for those <18 years old. Eligible participants were ages 14–21, met DSM-5 criteria for cannabis use disorder within the last 30 days, expressed interest in cannabis use disorder treatment, and submitted a urine sample positive for cannabinoids (> 50 ng/mL). Individuals currently enrolled in cannabis use disorder treatment, with moderate or severe substance use disorders aside from cannabis or nicotine/tobacco, with current (past 30 days) or planned synthetic cannabinoid use, pregnant or lactating, currently prescribed carbamazepine or nitroglycerin, with seizure disorder or uncontrolled severe asthma, or with acutely unstable medical or psychiatric disorders were excluded.

Participants self-reported baseline motivation, readiness, and confidence to quit using cannabis (all on a 1–10 scale, with 1 = “not” and 10 = “extremely”) and were randomized in 1:1 ratio to N-acetylcysteine or placebo, stratified by age (≤18 versus ≥19) and by nicotine use status (assessed via Clinical Laboratory Improvement Amendments of 1988 [CLIA]-waived point-of-care urine cotinine test, with cutoff of <50 ng/mL signifying a cotinine-negative sample and thus categorizing into the non-nicotine-use group). A stratified random block design was utilized with random block sizes of 4 and 6. The randomization schedule was developed by the study statistician prior to initiation of enrollment, using a blinded allocation (A/B), and the investigational pharmacy randomly assigned active NAC and placebo treatment to A/B assignments. Participants, clinicians, and study personnel were blind to treatment allocation throughout the study.

### Procedures

United States Pharmacopeia (USP) grade N-acetylcysteine powder was encapsulated in 600 mg quantities (two 600 mg capsules per dose). Matched placebo capsules were also prepared. All capsules were packaged and dispensed in blister packs, with individual labels for time/date of each dose. Participants were instructed to take two capsules (1200 mg) twice daily (total of 2400 mg per day), in approximately 12-h intervals; in the event of issues with tolerability, dose adjustments in increments of 600 mg were permitted at the discretion of the study medical clinician. Text messages prompted participants at the scheduled time for each dose, including a secure link for participants to upload a video recording of their medication-taking; study personnel reviewed participants’ uploaded videos to confirm adherence [14].

All participants received brief (typically <10 min) weekly medical clinician-delivered medical management and non-manualized skills-based cannabis cessation counseling (designed to match the intervention provided in the prior youth N-acetylcysteine trial, and to mimic what may be feasibly conducted in a busy clinical practice setting). The study, which included a hybrid of in-person and virtual visits, was conducted via a dedicated research clinic at the Medical University of South Carolina in Charleston, South Carolina.

### Outcome measures

Urine cannabinoid testing at baseline, during weekly visits, and at post-treatment follow-up visits, was conducted as the primary biological measure of cannabis use. Participants self-reported cannabis use throughout the study via mobile technology-delivered daily surveys, including quantification of daily cannabis and other substance use [14]. Missing daily substance use data were filled via Timeline Follow-Back-like procedures at study visits [15].

Weekly urine samples were tested qualitatively with CLIA-waived point-of-care cannabinoid tests (cutoff of <50 ng/mL signifying a cannabinoid-negative urine sample) and sent to the laboratory for quantitative cannabinoid and creatinine testing to allow for evaluation of creatinine-normalized cannabinoid levels [16, 17]. For virtual visits, necessitated as an option due to COVID-19 related restrictions to in-person visits, qualitative urine cannabinoid tests were conducted remotely but laboratory quantification was not performed.

Primary efficacy was assessed as self-reported abstinence from cannabis use confirmed by urine cannabinoid testing (<50 ng/mL) during the 12 weeks of treatment, measured at weekly study visits. In addition to abstinence, weekly proportion of days using cannabis (frequency) and grams of cannabis used per using day (amount) were compared between study treatment groups [18].

Adverse events were assessed for severity and relatedness to study treatment by the medical clinician at all visits and coded in Medical Dictionary for Regulatory Activities (MedDRA) terminology by body system.

Study personnel reviewed participants’ uploaded medication-taking videos to confirm adherence; as part of medical management, the medical clinician addressed medication adherence during weekly visits throughout treatment. Adherence was assessed as the percentage of video-verified doses compared with the expected number of doses taken, summarized at each weekly visit (range 0–100%). A participant was considered medication compliant when taking at least 80% of prescribed doses.

### Statistical analysis

A sample size of 67 participants per treatment group would provide 80% power with two-sided α = 0.05 to detect a group difference on the primary endpoint (proportion of negative urine cannabinoid tests); accounting for an anticipated 30% attrition rate, a sample size of 96 per group was deemed adequate for statistical power.

Participant baseline characteristics found to be significantly associated with cannabis use outcomes were included as covariates in adjusted model development. Self-reported 7-day point prevalence abstinence, cannabis use days, and cannabis use amounts were summarized at each weekly study visit as well as follow up visits. The main effect of N-acetylcysteine on negative weekly urine cannabinoid tests was assessed with a repeated measure log-linear regression using a general estimating equations framework (GEE). Models were computed using design covariates, including study treatment assignment, visit week, baseline cannabis use rates, and characteristics utilized in the stratification at randomization (age, urine cotinine resulting indicating nicotine use status). Working correlation structures were independently compared using the quasi-likelihood under the independence model criterion statistic [19]. All randomized participants were included in the primary analysis and assessed (1) using all available data and (2) with participants deemed non-abstinent at any missed visit (drop-out/loss-to-follow up included). Model based means were used to construct the pairwise comparisons of treatment groups. In addition to the longitudinal analysis of negative urine cannabinoid tests and 7-day point prevalence abstinence rates during treatment, rates were compared between treatment groups at each post-treatment follow-up visit using logistic regression models. Summary results are presented as means and standard deviations, while model-based results are presented as risk ratios (RR) and associated 95% confidence intervals. Secondary cannabis use outcomes were (1) percentage of days using cannabis (frequency) and (2) grams of cannabis per using day (amount) between study visits during treatment, and were compared between study groups using linear mixed effects regression models. Assumptions of residual normality were assessed using QQ plots, and when deviations from normality were determined, data transformations were made (e.g. natural logarithm, square root). In addition to the primary analysis, modifying effects of sex assigned at birth on treatment efficacy were examined. When significant, stratified treatment efficacy estimates were estimated.

Penetration of the medication blind was assessed at the end of study treatment. Between groups assessment of blinding efficacy was conducted using Pearson’s Chi-Square test statistic, and data are reported as proportions correctly identifying their actual treatment assignment.

Treatment emergent adverse events are reported as the total number of events and frequencies for the whole cohort, as well as stratified by treatment assignment for all events that occur during study treatment.

Adherence (proportion of participants taking ≥80% of medication doses) was compared between randomized treatment groups using generalized linear mixed effects models with outcome specific distributions (logistic). Overall group differences, as well as differential adherence over time, were assessed through inclusion of a treatment group factor, a linear time factor, and the appropriate interaction.

All analyses were conducted using SAS version 9.4 (SAS Institute, Cary, NC, USA).

## Results

### Baseline characteristics and trial retention

**Fig. 1:**
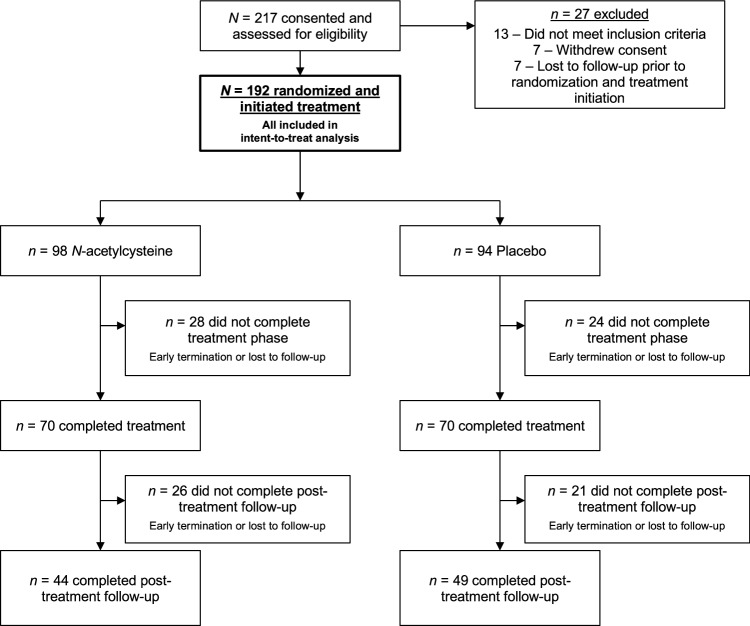
Recruitment and enrollment flowchart. Summary of participant engagement at all stages of the trial.

Participants were enrolled and data collected between August 2017 and January 2024. Among 217 participants consented and assessed for inclusion, 192 were randomized and initiated treatment (N-acetylcysteine *n* = 98 and placebo *n* = 94), and 140 attended the week 12 end-of-treatment visit (72.9%) (Fig. 1). Retention to end of treatment was similar between groups (N-acetylcysteine 71.4%, 70/98 and placebo 74.5%, 70/94). Of the 2304 possible weekly treatment visits (192 participant × 12 weekly visits), 1548 (67.2%) urine cannabinoid test results (1061 quantitative from in-person visits and 487 qualitative-only from virtual visits) and 1785 (77.5%) self-reported cannabis use summary measures were available for analysis. Further, 110 (57.3%) participants had data available at the 16-week follow-up and 93 (48.4%) at the 26-week follow-up visits. Demographic, clinical, and substance use history variables were summarized for the entire randomized cohort as well as stratified by randomized treatment assignment (Table 1). Participants were on average 19.2 years old (SD = 1.5), primarily female (52.6%), and white (78.5%); 14.1% were Hispanic and 10.4% were Black. In the 30 days prior to initial assessment, participants averaged 23.6 (SD = 7.6) cannabis use days. Additionally, 87.0% of participants had at least 1 alcohol use day and 71.4% used nicotine products; those who used nicotine averaged 17.4 nicotine use days in the 30 days prior to baseline (SD = 12.0).

**Table 1:** Demographics and clinical characteristics. Data are shown as means and associated standard deviations for continuous characteristics and count and percentages for categorical characteristics.

	N included	Overall		N-acetylcysteine		Placebo	
Age	192	19.2	1.5	19.1	1.3	19.2	1.7
Sex	192						
Male		91	47.4%	43	43.9%	48	51.1%
Female		101	52.6%	55	56.1%	46	48.9%
Race	192						
White		151	78.6%	78	79.6%	73	77.7%
Black		20	10.4%	9	9.2%	11	11.7%
Asian		2	1.0%	1	1.0%	1	1.1%
More than one race		15	7.8%	7	7.1%	8	8.5%
Prefer not to report		4	2.1%	3	3.1%	1	1.1%
Ethnicity	192						
Hispanic		27	14.1%	16	16.3%	11	11.7%
Cannabis use disorder severity	190						
Mild		24	12.6%	11	11.2%	13	14.1%
Moderate		36	19.0%	15	15.3%	21	22.8%
Severe		130	68.4%	72	73.5%	58	63.0%
Positive Baseline Cotinine Test	192	104	54.5%	54	55.1%	50	53.2%
Nicotine Use Days (any product)^a,b^	137	17.4	12.0	17.7	12.2	17.1	12.0
Cigarette Use Days^a,b^	54	9.0	9.6	7.9	7.9	10.2	11.2
E-Cigarette Use Days^a,b^	110	17.6	12.6	18.0	12.9	17.1	12.3
Alcohol Use Days^a,b^	167	5.5	4.2	5.2	3.8	5.9	4.5
Cannabis Use Days^a^	190	23.6	7.6	24.5	6.6	22.8	8.5
Urine Cannabinoids (ng/mL)^c^	150	764	1078	913	1181	597	929
Urine Creatinine (ng/mL)^c^	150	140	90	156	90	122	87
Cannabinoids to Creatinine Ratio^c^	150	7.7	11.8	8.2	12.6	7.0	11.1
Motivation, Readiness, and Confidence to Quit^d^	191						
Motivation		5.8	2.1	5.8	2.2	5.7	2.0
Readiness		5.3	2.4	5.2	2.5	5.4	2.3
Confidence		6.4	2.4	6.3	2.4	6.6	2.4

^a^Of the 30 days preceding screening assessment.

^b^among those with any self-reported use.

^c^quantitative urine cannabinoids and creatinine not available for participants completing initial assessment virtually due to COVID-related restrictions on in-person visits.

^d^participant self-rated on 1 (“not”) to 10 (“extremely”) scale.

### Baseline correlates of study outcome

Higher baseline cannabis use days (RR = 0.81 95% CI: 0.86, 0.91; *p* = 0.002) as well as daily cannabis use (yes/no) were both negatively associated with abstinence during treatment (RR = 0.18 95% CI: 0.09, 0.35; *p* < 0.001); baseline nicotine use status (RR = 0.29 95% CI: 0.13, 0.63; *p* = 0.01) was associated with decreased probability of weekly cannabis abstinence during treatment. Higher self-reported readiness (RR = 1.20 95% CI: 1.03, 1.39; *p* = 0.021) and confidence to quit (RR = 1.38 95% CI: 1.13, 1.68; *p* = 0.002) were significantly associated with higher likelihood of study abstinence, while baseline motivation was not (RR = 1.17 95% CI: 0.96, 1.42; *p* = 0.13). Sex assigned at birth, age, race, nicotine and alcohol use frequency, age at initiation of self-defined regular cannabis use, any prior cannabis quit attempts, self-defined regular e-cigarette use, and cannabis use disorder severity were not associated with study abstinence (all *p* > 0.05).

### Primary cannabis abstinence outcomes

**Fig. 2:**
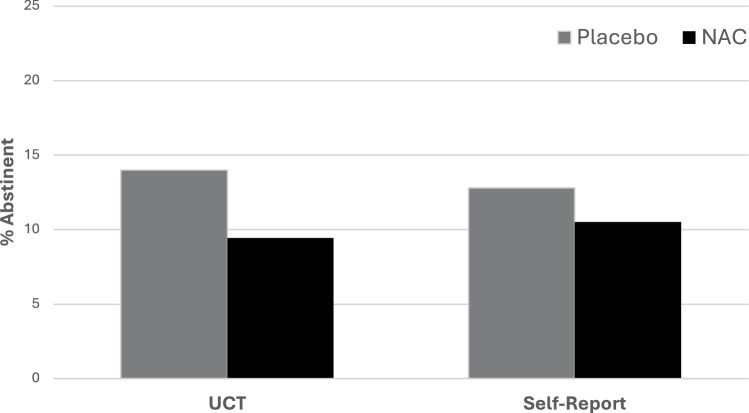
Abstinence rates stratified by treatment assignment. Data are shown as the percentage of negative urine cannabinoid tests (UCT) and self-reported 7-day point prevalence abstinence measured during 12 weeks of study treatment. NAC = N-acetylcysteine.

During study treatment, 182 (11.8%) urine samples were negative for cannabinoids (N-acetylcysteine 72/763, 9.4%; placebo 110/785, 14.0%) (Fig. 2). In design adjusted models (baseline cannabis use days, age, nicotine use status), there was no statistical difference in the rate of negative urine cannabinoid tests between N-acetylcysteine and placebo participants (RR = 0.93, 95% CI =0.53, 1.64; *p* = 0.80). When imputing missing data to positive for urine cannabinoids, results were consistent with available data (RR = 0.85, 95% CI = 0.46, 1.55; *p* = 0.59). At the 16-week follow-up visit, *n* = 72 of 192 randomized participants had urine cannabinoid data available with 10 negative urine cannabinoid tests; 5 in the N-acetylcysteine group (5/34, 14.7%) and 5 in the placebo group (5/38, 13.2%). Similarly at the 26-week follow up time point, *n* = 85 participants had available urine data with 15 negative urine tests; 6 in the N-acetylcysteine group (6/38, 15.8%) and 9 in the placebo group (9/47, 19.2%) (overall RR = 0.94, 95% CI: 0.39, 2.24, *p* = 0.88).

There was no evidence that participant sex assigned at birth modified treatment efficacy for urine cannabinoid test results (*p* > 0.70). Baseline confidence to quit showed evidence of differential relationship between the N-acetylcysteine and placebo participants (confidence × treatment interaction; χ^2^_1_ = 6.2; *p* = 0.013) with a significant association between higher confidence and increased abstinence in the participants randomized to receive N-acetylcysteine (RR = 1.66 95% CI: 1.40, 2.00; *p* = 0.001) but non-significant in participants randomized to receive placebo (RR = 1.08 95% CI: 0.94, 1.24; *p* = 0.27). A similar but statistically non-significant result was seen in baseline readiness to quit (readiness × treatment interaction; χ^2^_1_ = 3.3; *p* = 0.07) with a significant association between higher readiness and increased abstinence in the participants randomized to receive N-acetylcysteine (RR = 1.24 95% CI: 1.08, 1.43; *p* = 0.002) but non-significant in participants randomized to receive placebo (RR = 1.08 95% CI: 0.94, 1.24; *p* = 0.26).

### Secondary cannabis use outcomes

During study treatment, 208 (11.7%) weekly self-reports were negative for cannabis use (N-acetylcysteine 93/888, 10.5%; placebo 115/897, 12.8%) (Fig. 2). Self-reported weekly abstinence taken at each study visit during treatment showed no difference between groups in design adjusted models (RR = 1.02, 95% CI = 0.63, 1.65; *p* = 0.93) or imputed models (RR = 0.96, 95% CI = 0.57, 1.62; *p* = 0.89). There was no evidence that participant sex modified treatment efficacy for self-reported weekly abstinence (*p* > 0.20).

**Fig. 3:**
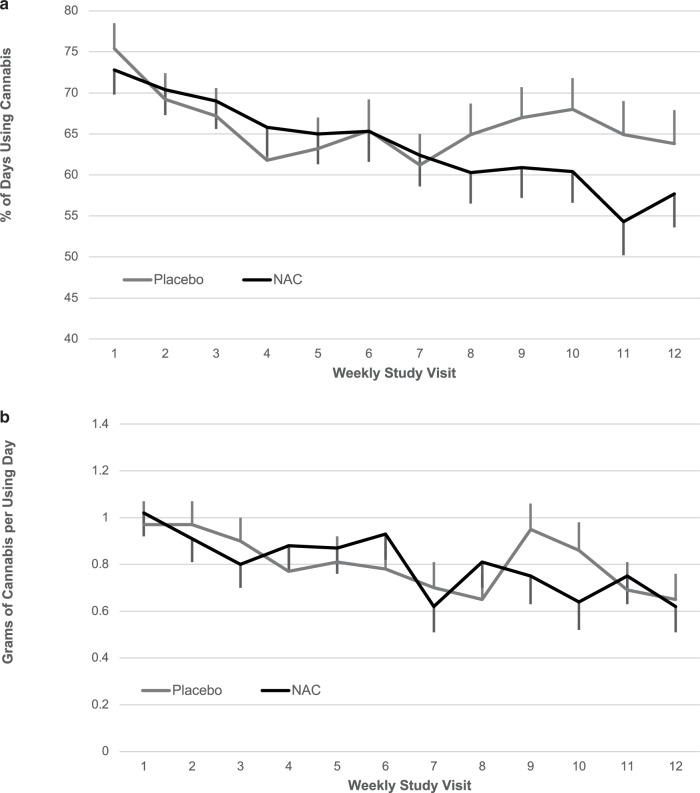
Self-reported cannabis use outcomes. Weekly average self-reported (**a**) percentage of cannabis using day (**b**) and grams of cannabis use per using day. Data are shown as model-based means and associated standard errors. NAC = N-acetylcysteine.

The mean percentage of cannabis use days decreased over time during study treatment (β = –0.01, SE = 0.003, F_1,144_ = 19.4, *p* < 0.001) but no differences were noted between study treatment groups (β = −0.01, SE = 0.039, F_1,180_ = 0.1, *p* = 0.78, Fig. 3a). Similarly, grams of cannabis used per using day decreased over time during study treatment (β = −0.03, SE = 0.009, F_1,667_ = 10.0, *p* = 0.002) but no differences were noted between groups (β = 0.00, SE = 0.08, F_1,315_ = 0.00, *p* = 0.99, Fig. 3b).

### Blinding efficacy

At the end of treatment, 128 participants responded to the penetration of the blind questionnaire; 53.2% (*n* = 33/62) of N-acetylcysteine participants and 69.7% (*n* = 46/66) of placebo participants correctly identified their treatment assignment (χ^2^_1_ = 3.7, *p* = 0.06).

### Safety/adverse events

Study medication was generally well tolerated. A total of 616 adverse events were reported by 159 participants (83%) across 12 weeks of study treatment (86/98 N-acetylcysteine participants and 73/94 placebo participants). Most reported events were considered “definitely not related to study treatment” (436/616, 70.8%) and only 14 (2.3%) reported events were considered severe (N-acetylcysteine 10/347 [2.9%] and placebo 4/269 [1.5%]). The most common adverse event category across both treatment groups was gastrointestinal (63/98 N-acetylcysteine and 37/94 placebo participants, χ^2^_1_ = 11.9, *p* < 0.001) followed by infections (mostly upper respiratory) (35/98 N-acetylcysteine and 38/94 placebo participants, χ^2^_1_ = 0.5, *p* = 0.50). Adverse events reported by ≥5% of participants in the overall sample and/or in either the N-acetylcysteine or placebo group are summarized in Table 2. Medication dose adjustment was made in 18 (9.4%) participants during treatment, with more made in the N-acetylcysteine group (14/98, 14.3%) than the placebo group (4/94, 4.3%; *p* = 0.02).

**Table 2:** Adverse events reported by ≥5% of participants in the overall sample and/or in either the N-acetylcysteine or placebo group. Data are shown as number and percentage of participants in each group.

		Overall (*N* = 192)	N-acetylcysteine (*n* = 98)	Placebo (*n* = 94)
Gastrointestinal disorders	Abdominal pain	8 (4.2%)	3 (3.1%)	5 (5.3%)
	Diarrhea	13 (6.8%)	5 (5.1%)	8 (8.5%)
	Dyspepsia	15 (7.8%)	10 (10.2%)	5 (5.3%)
	Gastroenteritis	7 (3.6%)	5 (5.1%)	2 (2.1%)
	Gastroesophageal Reflux Disease	17 (8.9%)	14(14.3%)	3 (3.2%)
	Nausea	48 (25.0%)	32 (32.7%)	16 (17.0%)
	Stomachache	11 (5.7%)	7 (7.1%)	4 (4.3%)
	Vomiting	25 (13.0%)	16 (16.3%)	9 (9.6%)
Infections and infestations	Sinusitis	9 (4.7%)	5 (5.1%)	4 (4.3%)
	Viral Upper Respiratory Tract Infection	50 (26.0%)	23 (23.5%)	27 (28.7%)
Metabolism and nutrition disorders	Decreased Appetite	16 (8.3%)	9 (9.2%)	7 (7.5%)
Nervous system disorders	Headache	41 (21.4%)	25 (25.5%)	16 (17.0%)
Psychiatric disorders	Anxiety	16 (8.3%)	5 (5.1%)	11 (11.7%)
	Depressed Mood	10 (5.2%)	2 (2.0%)	8 (8.5%)
	Insomnia	32 (16.7%)	18 (18.4%)	14 (14.9%)
	Irritability	12 (6.3%)	7 (7.1%)	5 (5.3%)
	Nightmares	10 (5.2%)	6 (6.1%)	4 (4.6%)
Respiratory, thoracic, and mediastinal disorders	Cough	9 (4.7%)	7 (7.1%)	2 (2.1%)

### Medication adherence

By treatment group, 75.3% (889/1176) of N-acetylcysteine and 80.3% (896/1116) of placebo weekly reports were categorized as adherent (RR = 0.98; 95% CI = 0.91, 1.05; *p* = 0.56), defined as taking ≥80% of doses. Further, medication adherence was not significantly associated with negative urine cannabinoid tests during treatment (RR = 0.98; 95% CI = 0.91, 1.05; *p* = 0.56).

## Discussion

In this randomized, placebo-controlled trial of N-acetylcysteine added to weekly medical clinician-administered brief cessation counseling and medical management for youth cannabis use disorder, demographic variables were well balanced between treatment groups, and participant retention and medication adherence rates indicated adequate power to test clinical outcomes. Across all participants, the percentage of cannabis using days and grams of cannabis used per using day decreased over time during study treatment; however, no difference in cannabis use reduction or cessation outcomes was noted between participants in the N-acetylcysteine and placebo groups. N-acetylcysteine was generally well-tolerated, differing from placebo only in the frequency of gastrointestinal adverse events; most events were rated as mild or moderate.

Efficacy findings differ from those of a similarly designed prior randomized, placebo-controlled trial of N-acetylcysteine for youth cannabis use disorder, which demonstrated more than doubled odds of negative urine cannabinoid tests during treatment in the N-acetylcysteine group compared to the placebo group [11]. While it is possible to interpret the present findings as a replication failure, the prior study’s design and execution differed from the present trial in two key ways: (1) inclusion of contingency management as a platform behavioral treatment to promote abstinence from cannabis and attendance at study visits, and (2) participant enrollment between 2009 and 2011, contrasted with the present trial’s enrollment spanning 2017–2023.

Contingency management is a robust behavioral treatment, providing salient reinforcers for evidence of desired behavior that may be particularly pertinent for youth who use cannabis; in the case of the prior N-acetylcysteine trial, this included an escalating schedule of monetary incentives for visit attendance and for negative urine cannabinoid tests [11, 20–22]. When treatment motivation is fleeting or limited, as often occurs with adolescents and emerging adults, contingency management may provide a key extrinsic reinforcer to motivate treatment engagement and bolster efforts to achieve substance abstinence. In the present study, baseline self-reported confidence and readiness—but not motivation—to quit cannabis were predictive of treatment success, indicating the importance of factors beyond motivation. Given prior evidence of synergy between contingency management and pharmacotherapy for youth substance use disorder treatment, the platform of behavioral incentives may have played a key role in facilitating the prior trial’s demonstration of N-acetylcysteine efficacy, contrasting with the present findings in the absence of contingency management [13].

The years between the prior and present N-acetylcysteine trials have seen substantial changes in cannabis-related policies across much of the US, associated with decreased perception of cannabis-related harm among youth [20]. Additionally, cannabis preparations have included higher concentrations of delta-9-tetrahydrocannabinol, the main psychoactive component in cannabis that drives its addictive potential [23]. While rates of youth cannabis use and cannabis use disorder have risen over that time span, recent findings highlight a paradoxical concurrent decrease in treatment admissions [24]. Though evidence is clear that youth-onset cannabis use disorder is impairing and associated with an array of adverse outcomes, in an environment of increasingly positive and permissive messaging related to cannabis, engagement in and response to cannabis use disorder treatment may be less robust [4]. Even with the present study’s treatment-seeking sample, cannabis abstinence rates during treatment were low, averaging below 15% across both treatment groups. Additionally, amid increases in co-occurring mental health symptoms among youth, coping motives for cannabis are increasingly common, suggesting increased clinical complexity [25, 26]. This indicates the need for enhanced approaches to tailoring treatment that are accessible and acceptable to youth and responsive to their clinical presentations [27, 28].

N-acetylcysteine’s role in substance use disorder pharmacotherapy remains unclear. While preclinical models indicate robust mechanistic and behavioral responses across substances, human laboratory and clinical trials have yielded a far less consistent picture [7, 8]. This translational gap may be owing to an array of factors, including details of trial design, such as inclusion criteria, dosing and duration, and embedded behavioral treatment; bridging these translational steps in substance use disorder pharmacotherapy development remains an overarching challenge to the field [29]. Additionally, given the complexity of biological, psychological, and social contributions to substance use disorders, other factors may maintain continued use even when a pharmacological intervention demonstrates a desired mechanistic response. This highlights the importance of developing integrated behavioral and pharmacological interventions that are complementary or synergistic in addressing factors that maintain addictive behaviors. It is possible that incentives to motivate change are necessary to complement N-acetylcysteine in promoting abstinence. Additionally, the finding that confidence and readiness to quit cannabis predicted treatment success in the present study—significantly so in the N-acetylcysteine group and not in the placebo group—indicates a potential pathway for tailoring N-acetylcysteine treatment for those most likely to respond.

Strengths of the present trial include its rigorous design and adequate sample size with similar representation across sexes, participant retention, and medication adherence to test clinical outcomes. Limitations include modest racial diversity in the enrolled participant sample, as well as reduced capacity for quantitative laboratory analysis of urine cannabinoids amid COVID-19 pandemic-related reliance on virtual visits; point-of-care qualitative testing is not considered as rigorous as quantitative laboratory analysis. Additionally, the study’s lack of mechanistic data on pharmacological target engagement significantly limits interpretation of null findings. Also of note, the two-group design (N-acetylcysteine versus placebo) without inclusion of contingency management elements allowed for only indirect comparison with the prior trial’s findings. Nonetheless, present findings indicate that N-acetylcysteine is not efficacious for youth cannabis use disorder when not paired with contingency management, highlighting the important role of behavioral incentives in facilitating N-acetylcysteine’s efficacy. Given the high prevalence and adverse outcomes associated with youth cannabis use disorder, more work is needed to develop and tailor behavioral and pharmacological treatments that are accessible to and effective for youth.

## Data Availability

The datasets generated during and/or analyzed during the current study are available from the corresponding author on reasonable request.
